# Optimizing Nanofluidic Energy Harvesting in Synthetic Clay‐based Membranes by Annealing Treatment

**DOI:** 10.1002/advs.202400233

**Published:** 2024-06-17

**Authors:** Yozelin Zavala‐Galindo, Guoliang Yang, Hanwen Zang, Weiwei Lei, Dan Liu

**Affiliations:** ^1^ Institute for Frontier Materials Deakin University Locked Bag 20000 Geelong VIC 3220 Australia

**Keywords:** 2D materials, clay‐based membranes, nanochannels, nanofluidics, osmotic power, salinity gradient

## Abstract

Nanofluidic energy harvesting from salinity gradients is studied in 2D nanomaterials‐based membranes with promising performance as high ion selectivity and fast ion transport. In addition, moving forward to scalable, feasible systems requires environmentally friendly materials to make the application sustainable. Clay‐based membranes are attractive for being environmentally friendly, non‐hazardous, and easy to manipulate materials. However, achieving underwater stability for clay‐based membranes remains challenging. In this work, the synthetic clay Laponite is used to prepare clay‐based membranes with high stability and excellent performance for osmotic energy harvesting. The Laponite membranes (Lap‐membranes) are stabilized by low‐temperature annealing treatment to effectively reduce the interlayer space, achieving a continuous operation under salinity gradients. Furthermore, the Lap‐membranes conserve integrity while soaking in water for more than one month. The output power density improves from ≈4.97 W m^−2^ on the pristine membrane to ≈9.89 W m^−2^ in the membrane treated 12 h at 300 °C from a 30‐fold concentration gradient. Especially, It is found that the presence of interlayer water to be favorable for ion transport. Different mechanisms are proposed in the Lap‐membranes involved for efficient ion selectivity and the states found with varying annealing temperatures. This work demonstrates the potential application of Laponite based nanomaterials for nanofluidic energy harvesting.

## Graphical abstract

The 2D‐nanofluidic device based on Laponite clay demonstrates excellent performance for osmotic energy harvesting after annealing treatment. The membrane treated at 300 °C displays superior selectivity for Na^+^ ions and high ion flux, leading to the maximum power density of 9.89 W m^−2^. This establishes the possibility of designing environmentally friendly Laponite‐based nanofluidic devices of facile fabrication and low cost.

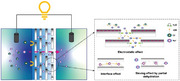

## 1 Introduction

The outgrowing demand for electricity has increased the need for new energy sources that can satisfy global requirements. Osmotic energy, also known as “blue energy,” has the potential to utilize the difference in electrolyte concentration found in the interfaces of fresh and salty water, salty and brine water, or wastewater streaming.^[^ ^1^ ^]^ Long‐term and sustainable energy harvesting from salinity gradients has been hindered by high operational costs, low power output, and low efficiency despite being in research since the 1960s.^[^ ^2^, ^3^, ^4^, ^5^ ^]^ Effective ion selectivity, high ionic conductivity, mechanical strength, and chemical and thermal stabilities are required in the osmotic energy harvesting membranes to make this technology feasible.^[^ ^6^, ^7^, ^8^, ^9^, ^10^ ^]^

Membranes based on 2D nanomaterials,^[^ ^11^ ^]^ such as graphene oxide,^[^ ^12^, ^13^ ^]^ layered‐double hydroxide (LDHs),^[^ ^14^, ^15^ ^]^ boron nitride,^[^ ^16^, ^17^ ^]^ MXenes,^[^ ^18^, ^19^ ^]^ and molybdenum disulphide,^[^ ^20^, ^21^ ^]^ have shown competitive performance as potential platforms for high power density and scalable membranes. Research is moving to environmentally friendly, highly abundant materials such as silk,^[^ ^22^, ^23^ ^]^ cellulose,^[^ ^24^ ^]^ and clays^[^ ^25^ ^]^ to reduce the cost of the membranes and promote sustainability. Smectite clays have advantages over other 2D nanomaterials to achieve lamellar assemblies that display surface‐charged governed proton transport behavior due to their natural layered morphology and chemical composition, and being non‐hazardous, chemically and thermally stable.^[^ ^26^ ^]^ In 2015, Huang et al. demonstrated effective surface charged‐governed transport in layered nanochannels from vermiculite clay.^[^ ^27^ ^]^ Recent works included 2D nanosheets exfoliated from varying clays as montmorillonite,^[^ ^28^ ^]^ kaolinite,^[^ ^29^ ^]^ and vermiculite.^[^ ^30^ ^]^ However, clay‐based membranes typically display poor mechanical properties and face poor underwater stability, hindering their continuous operation in nanofluidic applications. Annealing treatment is a primary post‐processing method to narrow the lamellar nanochannels and improve stabilization.^[^ ^6^ ^]^ However, the performance of reported pristine and annealed clay‐based membranes in osmotic energy harvesting is unremarkable to satisfy practical applications.

Natural clays such as montmorillonite and vermiculite vary in composition and properties according to their geological context of origin. Widely available and inexpensive raw materials, clays are rarely found pure. Residual metals and organic matter can cause unpredictable effects in advanced applications, such as variations in the chemical composition that consequently cause charge unbalances.^[^ ^31^, ^32^ ^]^ Synthetic clays can be produced in abundance, at a low price, and with controllable morphology, composition, and purity grade. Laponite is one of the most used synthetic clays in industrial applications,^[^ ^33^ ^]^ so studying derived nanomaterials is attractive in emerging applications. In this work, the synthetic clay Laponite was selected as the base material for synthesizing clay‐based membranes. Laponite is a synthetic magnesium silicate homologous to natural and rare occurring hectorite and similar in structure to montmorillonite.^[^ ^32^, ^34^ ^]^ Laponite nanomaterials have been previously used in ion‐related applications in composites^[^ ^35^, ^36^ ^]^ and nanofillers^[^ ^37^, ^38^ ^]^ and as coating materials for enhanced battery applications.^[^ ^39^, ^40^, ^41^ ^]^ Nevertheless, to the best of our knowledge, no other publication has synthesized free‐standing Laponite‐based membranes for osmotic energy harvesting. Therefore, it is worth exploring the synthesis of Laponite nanosheets (Lap‐nanosheets) and their reassembled membranes.

In this work, we demonstrated the impact of low‐temperature annealing treatment on the membrane structure and its influence on the characteristics of pristine Laponite membranes (Lap‐membranes). We characterized the effects of the treatment temperature and the different hydration states that arise. We found the optimal balance between ion selectivity and ion flux occurred in the treated membrane at 300 °C exhibiting a high output power density of >9 W m^−2^. Consequently, we proposed an insight into the different mechanisms influencing high selectivity related to multiple electrostatic and steric effects. Simultaneously, we explore the role of the small aspect ratio Lap‐nanosheets to achieve an effective performance for salinity gradient energy harvesting.

## 2 Results and Discussion

### 2.1 Nanosheets Characteristics

**Figure 1:**
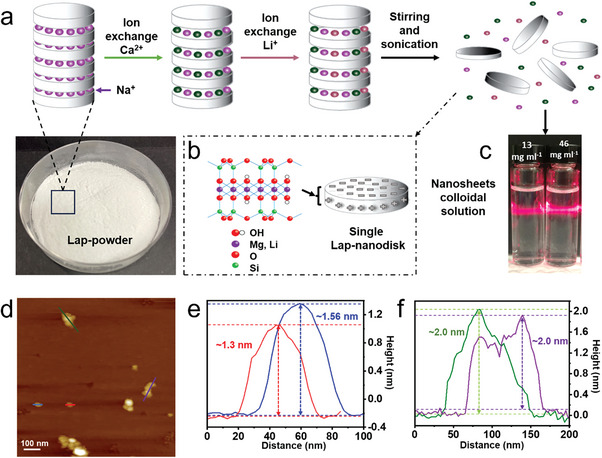
a) Schematic representation of the Laponite exfoliation process. b) Theoretical morphology and chemical structure of Laponitenanodisks. c) Photos of Laponite dispersion with the Tyndall effect. d) AFM of exfoliated Lap‐nanosheets: e) single layer and f) multilayer nanosheets.

**Figures** **1**a and S1 (Supporting Information) show the exfoliation process for Lap‐nanosheets. The starting Laponite powder is produced on a large scale and available in multiple grades. In Laponite (formula Na_0.7_^+^[(Si_8_Mg_5.5_Li_0.3_)O_20_(OH)_4_]_0.7_^−^), lithium ions substitute magnesium, giving the surfaces a permanent negative charge balanced by interchangeable sodium cations in the interlayer space (Figure 1a,b). Single Laponite nanodisks possess an ideal structure with a diameter of 25 nm and single‐layer thickness of 0.9–1 nm.^[^ ^31^, ^42^ ^]^ (Figure 1b). The edges of Laponite nanodisks have hydroxyl functional groups that can be protonated depending on surrounding pH.^[^ ^43^, ^44^, ^45^ ^]^ The different surface charges of the face and edge and the small size of the individual nanodisks promote the formation of a polymeric‐like network commonly known as the house of cards.^[^ ^46^, ^47^, ^48^ ^]^

To increase the surface area of commonly small Laponite nanodisks and to avoid undesirable interaction, a top‐down approach by multistep ion exchange processes was adopted (Figure 1a). The order of the exchangeable ions (Ca^2+^, Li^+^) was optimized so that the yield was maximized while avoiding aggregation and spontaneous gelation. Supernatants of exfoliated nanosheets have an average zeta potential of −30.5 mV at pH 8, as obtained from exfoliation, which provides stable and transparent colloidal solutions that exhibit a strong Tyndall effect (Figure 1c; Figure S2, Supporting Information).

Figure 1d displays the morphology of the exfoliated nanosheets that follow a polydisperse flake‐like structure where larger sheets possess lateral dimensions of more than 40 nm (Figure 1e,f; Figure S3, Supporting Information). The thickness of a single layer lies between ≈1 and 1.3 nm, which demonstrates single‐layer exfoliation (Figure 1e,f). Multilayered nanosheets are expected in the samples due to electrostatic interactions between the negative faces and the remaining cations on the surface.^[^ ^26^ ^]^

### 2.2 Reassembly Characteristics

**Figure 2:**
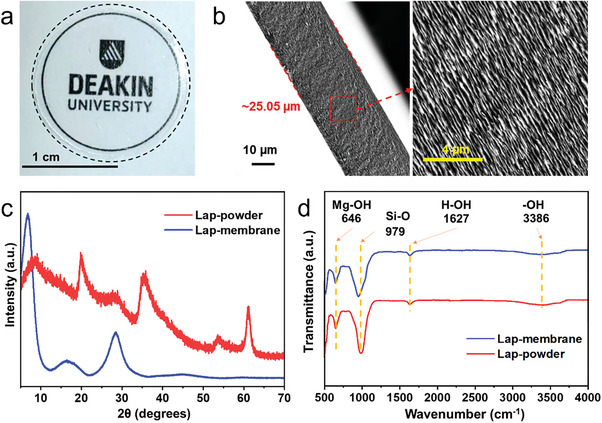
a) Photo of a free‐standing Lap‐membrane. b) SEM cross‐sectional view. Membrane possesses and approximate thickness of ≈25 µm. Inset: enlarged view of lamellar microstructure. c) XRD of bulk Lap‐powder and 2D‐clay‐based reconstructed membrane. d) FT‐IR spectra of bulk powder and reassembled Lap‐membrane.

Lap‐membranes were prepared by vacuum filtration. Free‐standing membranes (**Figure** **2**a) can be easily peeled from the Celgard substrate but do not possess high flexibility in comparison with similar reconstructed membranes from other clays such as Montmorillonite^[^ ^28^ ^]^ and Vermiculite,^[^ ^49^ ^]^ possibly related to the smaller aspect ratio of the nanosheets and the weak electrostatic forces in the interlayers.^[^ ^31^ ^]^ As shown in Figure 2b and inset, the cross‐section of the Lap‐membrane shows the clear formation of layered nanochannels with an ordered self‐assembled lamellar microstructure. The thickness of the pristine Lap‐membrane is ≈25 µm. The thickness of the membranes is adjusted by the concentration and volume of the colloidal dispersion used for filtration.

The pristine Laponite powder (Figure 2c) shows wide peaks at 8° {001}, 20° {100}, 28° {005}, 35° {110}, 53°{310}, 61° {060} characteristic of its amorphous nature.^[^ ^50^ ^]^ After ion exchange and reassembly of the Lap‐membranes, the first three visible diffraction peaks shifted to lower angles, which is an indication of successful exfoliation and the increment of interlamellar spacing. An intense peak can be identified in the Lap‐membranes before 10°, corresponding to the interplanar spacing between the nanosheets. However, the diffraction angle is slightly variable between samples due to the level of retention of absorbed water during the vacuum filtration stage (Figure S4a, Supporting Information).

Hydroxyl functional groups are visible in the FT‐IR spectra (Figure 2d; Figure S4b, Supporting Information). The main visible characteristic peaks of Laponite are at 646 cm^−1^ corresponding to a Mg─OH bending vibration and 979 cm^−1^ for Si─O stretching vibration. FT‐IR pattern displays a broadband at 3386 cm^−1^ attributed to ─OH vibrations of adsorbed water molecule (intermolecular bonding) and a sharp band at 1627 cm^−1^ assigned to H─OH bending.^[^ ^51^, ^52^ ^]^ The most significant difference is in the Si─O stretching vibration peak, which has an obvious blueshift (from 979 to 946 cm^−1^) that can be related to the decrease in the particle size and the reduction in the strength of the interlayer bonding because of the exfoliation process.^[^ ^53^, ^54^ ^]^ It is well known that clay‐based membranes are susceptible to delamination in water due to the interlayer's weak electrostatic forces and the clays’ high hydrophilic nature (Figure S5, Supporting Information). When soaked in water, the Laponite membranes were rapidly swollen and redispersed (Figure S6, Supporting Information); however, following annealing treatment, membranes exhibited stability for several weeks.

### 2.3 Annealing Treatment

Annealing is a commonly used post‐process for clay‐based membranes to remove physisorbed water and counteract their weak water stability.^[^ ^30^, ^55^ ^]^ High thermal stability of the Lap‐membranes is well translated during reassembly, as observed in the thermogravimetric analysis (Figure S7, Supporting Information). As shown in Figures S7 and S8 (Supporting Information), the evaporation of absorbed water can be observed as a rapid weight loss before 200 °C but does not imply a significant narrowing of the interlayer space (Figure S9, Supporting Information). With the increasing temperature, the Lap‐membrane experiences a slow weight loss until 650 °C, where it undergoes an exothermic phase transition exhibited by synthetic magnesium silicates (Figure S8, Supporting Information).^[^ ^51^, ^56^ ^]^ After the phase transition, the membrane has a significant structural change, becoming very soft and hard to handle (Figure S10, Supporting Information). Therefore, the annealing process was methodically characterized at 200 °C, 300 °C, and 400 °C to investigate if the Lap‐membrane exhibits significant properties changes that influence their permeability and selectivity characteristics for osmotic energy harvesting. The treated membranes were named X‐membrane (*X* = 200, 300, and 400) according to the annealing temperatures of 200 °C, 300 °C, and 400 °C.

**Figure 3:**
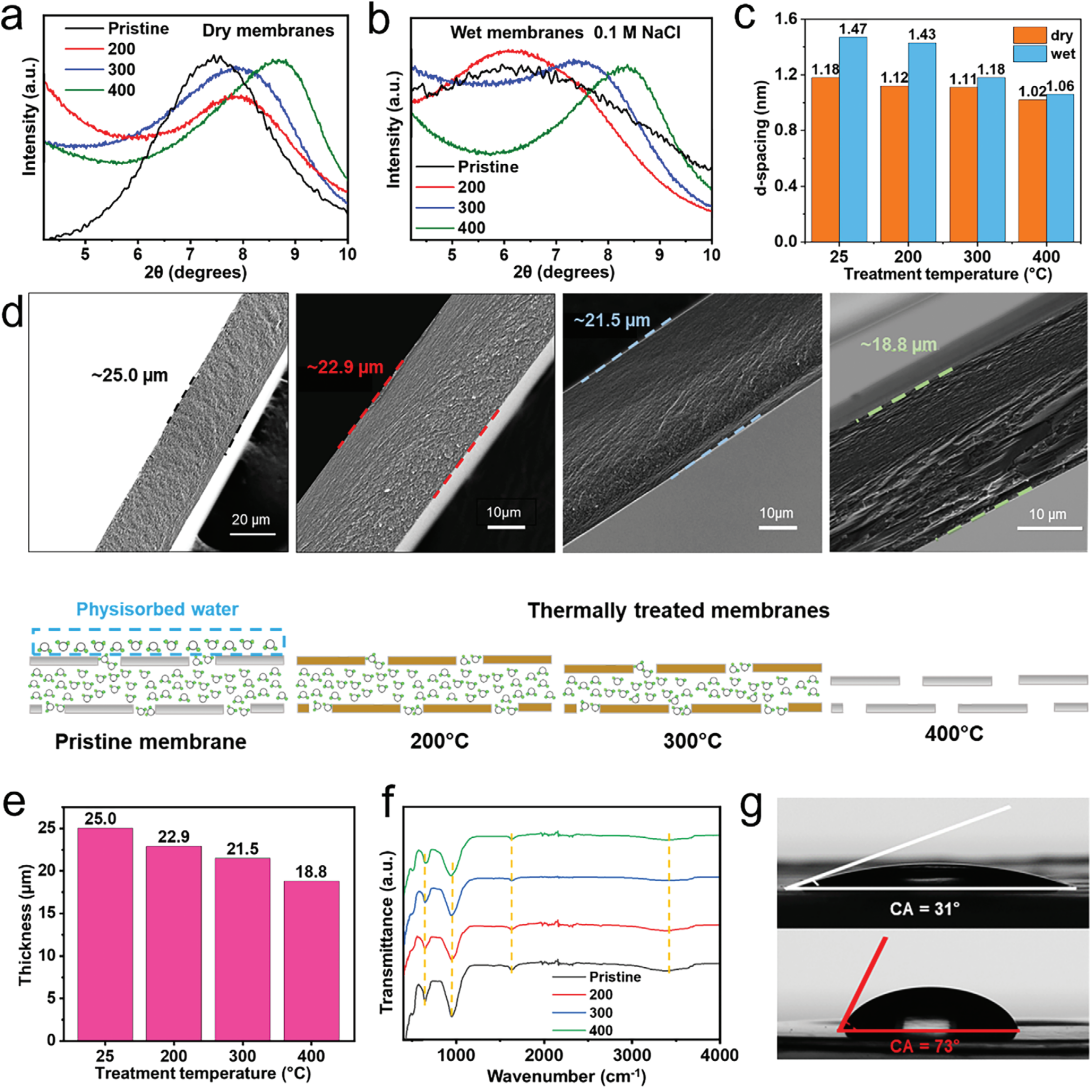
Characterization of pristine membrane in comparison to thermally treated membranes a) Dry‐state XRD. b) Wet‐state XRD. c) Comparison of d‐spacing changes. d) SEM cross‐sectional view. Thickness of membranes decreased after thermal treatment due to the removal of interlayer water. e) Thickness comparison between membranes. f) FT‐IR spectra of Lap‐membranes. g) Water contact angle before (top) and after (bottom) thermal treatment at 200 °C.

Low‐temperature annealing treatment can enhance the stability of clay membranes due to the Hoffman−Klemen effect (also known as the lithium self‐diffusion anomaly).^[^ ^57^, ^58^, ^59^ ^]^ In smectites, particularly lithium‐saturated smectites, low‐temperature annealing (<500 °C) can cause migration of interlayer lithium to octahedral spaces, inducing a charge change unbalanced.^[^ ^60^, ^61^ ^]^ Li^+^ can move vacant spaces in the octahedral or tetrahedral interlayer space, expanding the layer and promoting the diffusion of more Li^+^ ions and water, which increases hydrophobicity and decreases swelling.^[^ ^60^, ^62^, ^63^, ^64^, ^65^ ^]^ To evaluate the effect of annealing treatment on the Lap‐membranes, we first characterized the change in the interlayer spacing and the swelling capacity of the membranes. **Figure** **3**a shows the XRD with the corresponding change in the interlayer spacing for the thermal treatments investigated. Furthermore, the treated membranes were soaked in a saline solution (0.1 m NaCl) to observe their swelling behavior (Figure 3b). The graph in Figure 3c summarizes the changes in the interlayer distance between the dry and wet state. As the temperature increases, absorbed water migrates out of the interlayer, allowing the narrowing of the nanochannels and the overall contraction in the thickness of the membranes (Figure 3d,e). The annealing treatment does not affect the lamellar structure of the membranes, which remains uniform. The 200‐membrane contains a significant amount of water in the interlayers favoring permeation. Therefore, swelling occurs in a similar dimension as the pristine membrane with a Δd ≈ 0.3 nm. However, restricted swelling can be observed in the 300‐ and 400‐membranes, where the change in the interlayer space gets reduced more than three times to Δd ≈ 0.07 and Δd ≈ 0.04 nm, respectively.

Interestingly, the transparent reassembled Lap‐membranes exhibit a color change after annealing at 200 °C and 300 °C, which is not reversible after rehydration, which indicates that the annealing treatment has some permanent effect on the treated membranes. On the other hand, the 400‐membrane is transparent as the pristine membrane, pointing to an intermediate structural state for the 200‐ and 300‐membranes that display distinctive optical properties (Figure S11, Supporting Information). No noteworthy transition is observable in the FT‐IR spectra (Figure 3f); however, in XRD (Figure S12, Supporting Information), besides the change in the d‐spacing mentioned above, a slight shift in the peak assigned to the (110) plane corresponding to the edges of the nanosheets is noticeable, supporting the idea of a structural difference in the layers. The change in the water contact angle in Figure 3g reflects the transition to a hydrophobic surface after the thermal treatment. Stabilization was successfully achieved, as it is shown in Figure S13 (Supporting Information). In contrast to the pristine membrane that rapidly disperses, treated membranes are stable in DI water for several days.

SEM and AFM characterizations of the surface morphology (Figure S14, Supporting Information) display a constant property. The surface is mostly smooth except for the 300‐membrane, which shows an apparent roughness compared to the other membranes. The 300‐membrane displays a more significant roughness of 10.1 nm, ≈5 nm larger than the other membranes. A more wrinkled morphology could be related to the collapse of the nanochannels during the removal of water due to the annealing process.^[^ ^66^ ^]^ Nevertheless, it could indicate the transitional state at the given temperature (300 °C).

Thermal stability is preserved after the annealing treatment (Figures S15 and S16, Supporting Information). Treated membranes follow the trend of non‐treated membranes when characterized by thermogravimetric analysis. Table S1 (Supporting Information) details the mass loss at the different zones previously identified (Figure S8, Supporting Information). Although the 400‐membrane seems to lose more mass than the 300‐membrane, the most significant loss occurs <150 °C. At this temperature range, the superficial physisorbed water evaporates. In zone two, where the interlayer water migrates, the membranes show a decreasing loss in agreement with the structural changes previously observed. Interestingly, the exothermic phase transition shifts to lower temperatures that could be related to the level of dihydroxylation occurring in the Lap‐membranes as a possible enthalpic change after the annealing process.

### 2.4 Ion Transport and Osmotic Energy Harvesting of 300‐Membranes

**Figure 4:**
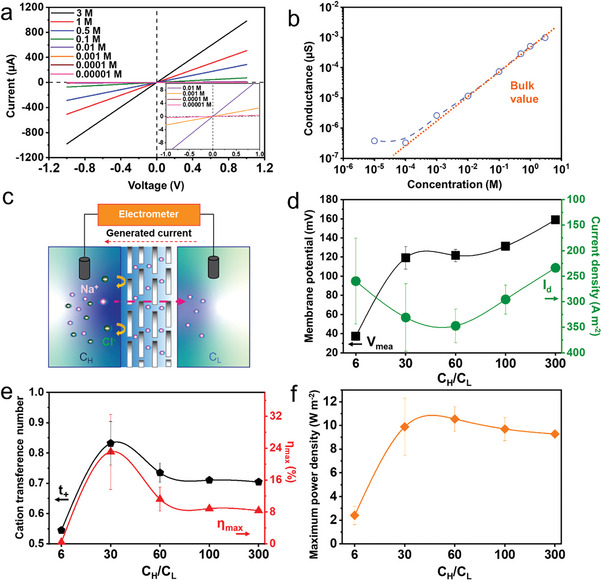
a) *I*–*V* curves through 300‐membrane. b) Ionic conductance of 300‐membrane c) Schematic diagram of ion transport measurement. Note: *C*_H_ and *C*_L_ denote the high and low concentration of the electrolyte respectively. d) Membrane potential and current density measured as a function of NaCl concentration gradient. *C*_H_ was fixed to 3 M, while *C*_L_ was varied for the corresponding fold (mean ± SD, *n* ≥ 2). e) Calculated cation transference number and efficiency (mean ± SD, *n* ≥ 2). f) Maximum power density (mean ± SD, *n* ≥ 2).

Membranes with a weight of 13 mg and thermally treated at 300 °C were utilized to investigate the ionic transport properties at the transitional state in NaCl electrolytes with concentrations from 10 µM to 3 M (**Figure** **4**a). Membranes were mounted in the middle of the H‐cell, and a pair of Ag/AgCl electrodes were placed in each side and connected to the electrochemical station. As Lap‐nanosheets are negatively charged, in the assembled nanochannels, Na^+^ cations will favorably diffuse, while Cl^−^ anions will be excluded. In Figure 4b, the measured ionic conductance follows a linear relationship in the high‐concentration region to gradually stabilize in the low‐concentration region (<1 mM), indicating the surface‐charge‐governed ion transport behavior. This behavior is supported by the d‐spacing of the nanochannels being smaller or close to the Debye length in the low concentration range.^[^ ^67^ ^]^ Ions migrate freely in the bulk salt solution, producing an ionic conductance proportional to the salt concentration (orange dashed line Figure 4b).

For the energy harvesting experiments (Figure 4c), the H‐cell was filled with the same volume of low (*C*_L_) and high‐concentration (*C*_H_) solutions for gradients (*C*_H_/*C*_L_) varying from 6 to 300 with a fixed *C*_H_ = 3M. The open‐circuit voltage (*V*_mea_) and the short‐circuit current (*I*_mea_) were recorded for each gradient and averaged from at least two measurements (Figure 4d). The open‐circuit voltage (*V*_mea_) and the short‐circuit current (*I*_mea_) comprise the potential and current across the membrane, as well as the redox contribution from the electrodes.^[^ ^68^, ^69^ ^]^ The membrane potential has a positive increment as the gradient increases from ≈37 to 158 mV. In contrast, the current density reaches a maximum at 60‐fold where *I*_mea_ ≈ 350 A m^−2^ (*V*_mea_ ≈ 121 mV) and then gradually declines.

Simultaneously, the *V*_mea_ gets away from the theoretical diffusion potential calculated by the Nerst equation for ideal cation selective membranes. The contribution from the redox potential is deducted from *V*_mea_ to effectively determine the diffusion potential (*E*_diff_) and the cation selectivity. In the case of complete cation selectivity, the transference number of cations *t*_+_ = 1, while *t*_+_ = 0.5 represents no cation selectivity and *t*_+_ = 0 total anion selectivity. At higher concentrations, the carriers available increase, which benefits the ionic flux; however, the low‐concentration side plays a significant role in the stability of the nanochannels, which reduces the ion selectivity as can be observed in the declined transference number of cations *t*_+_ and the efficiency *ƞ*_max_ in Figure 4e. The maximum power density of the nanofluidic device reaches a value of 10.4 W m^−2^, which shows the potential of Lap‐membranes for osmotic energy harvesting (Figure 4f).

**Figure 5:**
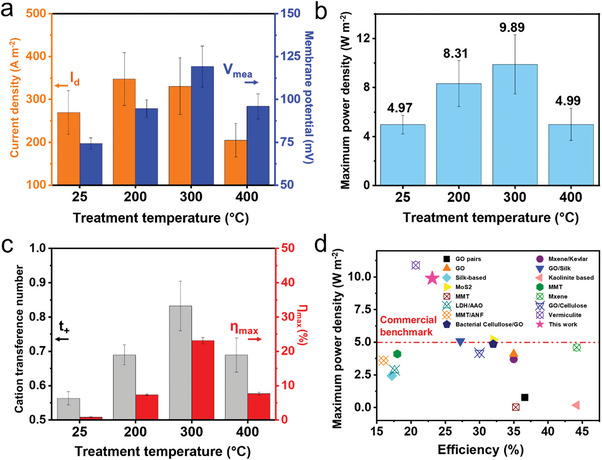
Energy conversion measurement under 30‐fold (*C*_H_/*C*_L_) for the pristine Lap‐membrane (Dried at room temperature 25 °C) and thermally treated membranes (200 °C, 300 °C, and 400 °C) a) Membrane potential and current density (mean ± SD, *n* = 3). b) Calculated cation transference number and efficiency (mean ± SD, *n* = 3). c) Maximum power density. (mean ± SD, *n* = 3). d) Comparison of power density from this work and other state‐of‐the‐art membrane‐based nanofluidics.^[^ ^12^, ^13^, ^14^, ^18^, ^21^, ^22^, ^23^, ^24^, ^29^, ^70^, ^71^, ^72^, ^73^, ^74^, ^75^ ^]^

Lap‐membranes treated at different temperatures were used to examine further the annealing treatment's influence on the reassembled membranes’ ion transport characteristics. A concentration gradient of *C*_H_/*C*_L_ = 30 with sodium chloride solutions *C*_H_ = 3M and *C*_L_ = 0.1 M was selected in a concentration range where the pristine membranes displayed stable operation. **Figure** **5**a displays the current density and membrane potential of the pristine Lap‐membrane and treated membranes, both having a noticeable improvement with the narrowing of the nanochannels during the annealing treatment at 200 °C and 300 °C. However, at 400 °C, the reassembled nanochannels have a very small d‐spacing of 1.06 nm (Figure 3c), which increases the resistance of the membrane directly impacting the current density, the ion selectivity and output power density (Figure 5b). The current density in the 200‐membrane and the 300‐membrane apparently benefits from the residual water in the interlayers which reduces the resistance of the membrane and favors ion transport; however, the smaller interlayer distance in the 300‐membrane favors the selectivity. Therefore, the 300‐membrane displays the best balance between current density and high cation selectivity, reaching a maximum t_+_ ≈ 0.83 and a ≈23% efficiency. The pristine membrane and the 400‐membrane display a similar maximum power density hindered by poor ion selectivity and high resistance, respectively (Figure 5c), while the 300‐membrane reaches the maximum value of 9.89 W m^−2^ with a high performance for osmotic energy harvesting (Figure 5d).

Figure S17 (Supporting Information) displays the long‐term energy generation of the 300‐membrane for the 30‐fold gradient of 0.1 and 3 M NaCl at neutral pH. Both the current and the power density (319.55 ± 30.70 A m^−2^ and 7.68 ± 0.79 W m^−2^, respectively) suffered from fluctuations related to the electrolyte. We replaced the electrolyte when both the voltage and the current measured had a notorious decrease. Nevertheless, the membrane potential was relatively stable and remained at 82% relative to the initial selectivity over 62 days, manifesting a long‐term stable structure of the 2D nanochannels.

The effect of weight or mass loading on the osmotic energy conversion performance (for the 30‐fold gradient of 0.1 and 3 M NaCl) of the 300‐membranes was further explored (Figure S18, Supporting Information). The output power density *P*_out_ and the internal resistance were investigated by connecting an external load resistor. The maximum output power density will appear when the internal resistance of the membrane is equal to that of the external load resistor.^[^ ^20^ ^]^ Figure S18 (Supporting Information) shows that the resistance of the reference 300‐membrane (13 mg) and the one with half the weight loading (6 mg) is similar, around ≈7 kΩ. However, the thinner membrane displays an increased current density, favoring the high output power density. On the contrary, with the increasing nanosheet content, the resistance increased to ≈10 kΩ, significantly impacting the power density. It is noticeable that the Lap‐based membranes' resistance is relatively small compared to other 2D materials,^[^ ^70^, ^76^ ^]^ favoring a high current density.

We also investigated the osmotic energy performance of the 300‐membranes in artificial seawater conditions (0.5 and 0.01 M NaCl) and hypersalinity (5 and 0.01 M NaCl) as shown in Figure S19 (Supporting Information). In the artificial seawater conditions, the membranes displayed an excellent efficiency of 27%, comparable to other 2D nanomaterials such as MXene and graphene oxide (Table S2, Supporting Information). In contrast, in the hypersalinity conditions, the selectivity was very low, due to the surface charge being screened by the high concentration of the electrolyte.^[^ ^77^, ^78^ ^]^

There are different factors influencing the performance of the thermally treated Lap‐membranes. Considering the small lateral size of the Lap‐nanosheets, the interconnected network of nanochannels could be modeled with multiple and less tortuous pathways that improve ion flux and increase the current density compared with traditionally larger nanosheets. While ionic transport usually benefits from thin membranes with shorter travel distances, we found that small nanosheets can have a similar effect even in micro‐thick membranes.^[^ ^79^ ^]^

**Figure 6:**
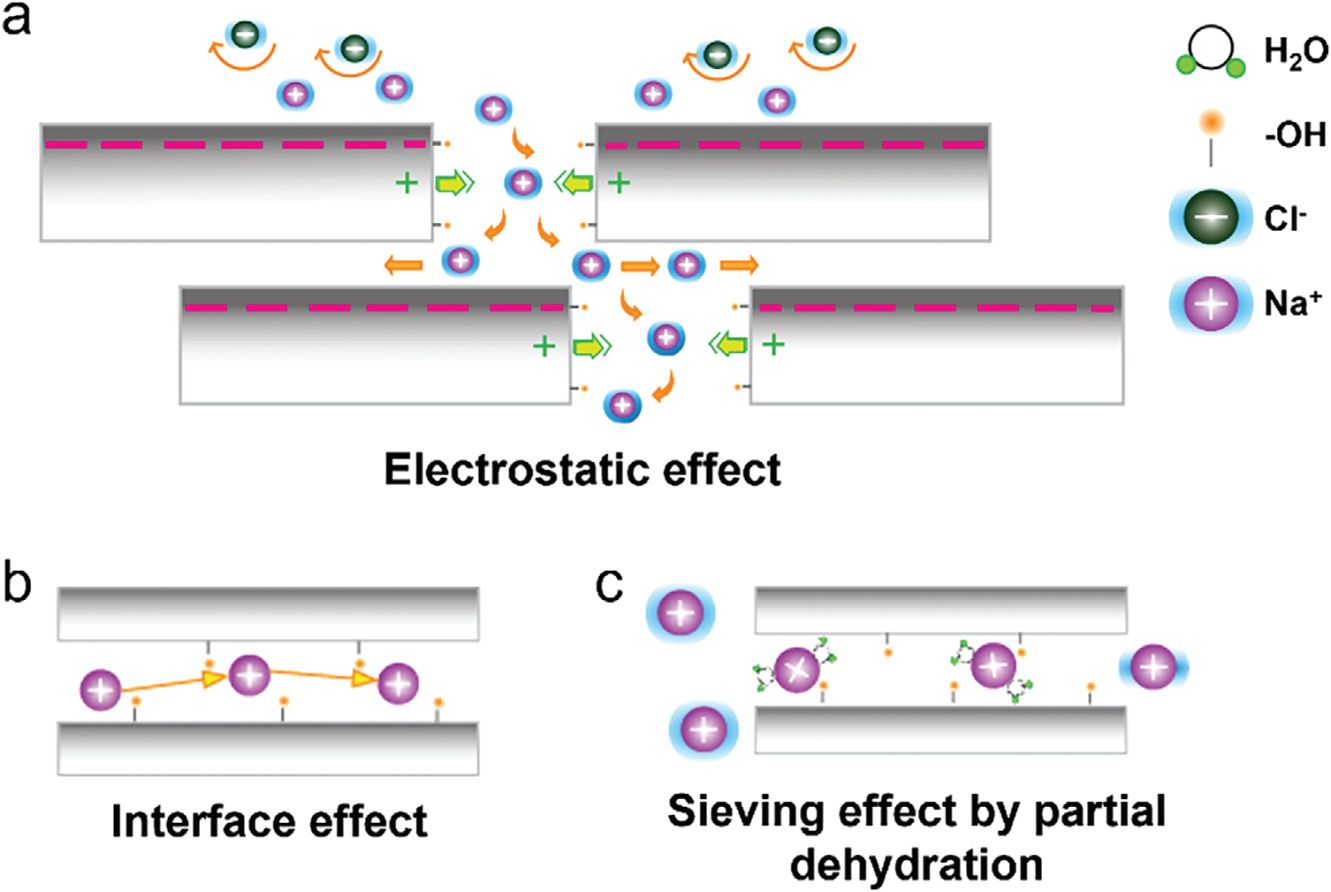
Graphic representation of ion transport phenomena in Lap‐membranes. a) Electrostatic effects and vertical pathway. b,c) Ion transport effects inside the nanochannels (Horizontal pathway).

There are multiple potential effects that favor ion transport inside the Lap‐nanochannels (**Figure** **6**). At the electrolyte interface, the steady negative surface charge of the faces favors the separation of charges and the preferential transport of cations (Figure 6a). The ion transport also benefits from the terminating ─OH functional groups that allow a fast transport in the nanoconfinement (Figure 6b).^[^ ^80^ ^]^ As observed in the FTIR spectra (Figure 3f), these functional groups remain relatively constant, ensuring ion selectivity. Furthermore, the broken edges of the Lap‐nanosheets include fixed moieties in the form of Si─OH, Mg─OH, and Li─OH.^[^ ^81^ ^]^ These amphoteric terminations provide the positive edges of the Lap‐nanosheets that could serve as “nano‐pumps” that impulse ions by repulsion, boosting their fast transport through the vertical path in the multilayer membrane (Figure 6a).

Note that the effective nanochannel height falls in the sub‐nanometer scale, but ion transport of hydrated ions is possible with partial dehydration, as observed in ZIF‐based and MXene membranes.^[^ ^80^, ^82^ ^]^ This difference in the interlayer distance could also produce a sieving effect (Figure 6c). In this regard, the remaining water in the 200‐membrane and 300‐membrane would be beneficial to keep the nanochannels with enough expansion to reduce the energy barriers.

## 3 Conclusion

In summary, we successfully demonstrate the fabrication and stabilization of Lap‐membranes for nanofluidic energy harvesting. The cationic Lap‐membranes show an ordered lamellar structure that prevails through the annealing treatment up to 400 °C. The nanochannel height decreases with the annealing temperature, restricting swelling compared with the pristine membrane. The membrane treated at 300 °C displayed optimal characteristics with good selectivity for Na^+^ ions and high ion flux, leading to the maximum power density of 9.89 W m^−2^, 200% better than the pristine membrane. We proposed three mechanisms that potentially influenced the performance of the Lap‐membranes. First, the small aspect ratio nanosheets are effective in creating short and fast pathways for ion transport despite the micro‐thickness of the membranes. Second, steric effects are due to the effective sub‐nanometer channels with partial dehydration of the ions. Finally, multiple electrostatic interactions originated from the negative surface charge, the functional ─OH groups on the surface, and simultaneously in the edges of the nanosheets. Therefore, this study suggests the possibility of designing Laponite‐based nanofluidic membranes with desirable characteristics such as environmentally friendly, low‐cost, safe, and facile fabrication.

## 4 Experimental Section

### Chemicals and Reagents

Laponite RD powder was kindly provided by IMCD Australia Pty. Ltd. (Manufacturer: BYK‐Chemie GmbH, surface area (BET) 370 m^2^ g^−1^, bulk density 1000 kg m^−3^ (20 °C), chemical composition: SiO_2_ 59.5%, MgO 27.5%, Li_2_O 0.8%, Na_2_O 2.8%, loss on ignition 8.2% as provided by manufacturer). The following salts and chemicals were purchased from Australia Sigma–Aldrich Pvt. Ltd. and used as received: lithium chloride (LiCl, ACS reagent, ≥99%), calcium chloride (CaCl_2_, anhydrous, granular, ≤7.0 mm, ≥93.0%), sodium chloride (NaCl, ACS reagent, ≥99.0%). Anodisc inorganic filter (AAO, 100 nm pore size and diameter of 25 mm) membranes as support were purchased from Whatman Co. and Celgard 2400 Polypropylene PP Battery Separator Film as substrates used as received.

### Synthesis of Lap‐Nanosheets

Lap‐nanosheets were synthesized by a multistep ion exchange top–down process. Normally 2.0 g of Laponite powder were dispersed in 200 mL of 1M CaCl_2_ solution and stirred at 850 rpm at 80 °C for 24 h. The suspension was vacuum filtrated before repeated centrifugation washing with deionized water and ethanol. The as‐obtained product was redispersed in 200 mL of 2 M LiCl solution to start the second cation exchange, as described previously. The sediment collected after centrifuge washing was then dispersed in water and the suspension was subjected to ultrasonification for 30 min. The supernatant slurry was obtained after 20 min centrifugation at 3500 rpm. Finally, reassembled nanofluidic channels into free‐standing membranes were achieved by means of vacuum filtration, where different volumes of the supernatant slurry were filtered through hydrophobic Celgard membranes as substrates and supported by AAO over the filtration device. Fixed volumes of Lap‐suspension were dried to estimate the concentration of the corresponding dispersion.

### Fabrication of Lap‐Membranes

Certain amounts of Lap‐nanosheets were vacuum‐filtrated on Celgard membranes. The effective filtration area was 3.1 cm^2^. The free‐standing membranes were peeled off from substrate after drying in ambient conditions. Annealing treatment on the dried Lap‐membranes was performed in a 1400C SANTE Dual Zone Tube Furnace for 12 h at 200 °C, 300 °C, and 400 °C in air as indicated. Unless otherwise specified, the total content of the membranes was 13 mg.

### Characterization

AFM images were obtained by a Bruker Multimode eight instrument. Morphology of Lap‐nanosheets and surface and cross‐section images of reassembled membranes were obtained and analyzed by SEM Zeiss Supra 55VP. X‐ray diffraction measurements were performed using an X‐pert Powder with Cu Kα radiation (λ = 1.54181 Å, 40 kV, and 30 mA). Optical transmittance spectra were recorded using a Varian Cary 300 UV–vis spectrometer. Mid‐infrared analyses of the original Laponite powder and reassembled Lap‐films were performed using a Bruker Invenio‐R FTIR spectrometer operating in Attenuated Total Reflectance (ATR) mode. Thermogravimetric analysis was achieved on a TA Q50 Thermogravimetric Analyze instrument, from room temperature to 850 °C, under oxygen flow (60 mL min^−1^) and nitrogen flow (60 mL min^−1^) at a heating rate of 10 °C min^−1^ Contact angle measurements of Lap‐membranes were performed using Attention Theta Flow Optical Tensiometer. ζ potential tests were performed with the use of a Zetasizer Nano ZS90 apparatus from Malvern.

d‐spacing calculation was done according to Bragg's Law (Equation 1):

(1) nλ=2dsinθ

where *n* is the order of reflection, *λ* is the wavelength, *d* is the interplanar spacing (d‐spacing), and the angle of reflection *θ*.

### Electrical Characterization

Ion transport through the Lap‐membranes was investigated with a homemade H‐cell. NaCl aqueous solutions were prepared as electrolytes with concentrations from 10^−5^ M to 3 M. For the ionic conductance characterization, a Lap‐membrane was fixed in the middle of the H‐cell and a pair of Ag/AgCl electrodes was placed on each side and connected to the electrochemical station (reference 600+, Garmy Co., Ltd) to record the *I–V* curves. Voltage input was varied between −1 and 1 V. The electric conductance *G* is calculated with Equation (2)^[^ ^83^ ^]^:

(2) $$G=\frac{I}{\Delta V}$$

where *I* is the ionic current generated under the electric potential drop ΔV.

For the osmotic energy harvesting measurements, the H‐cell was filled with the same volume of low and high‐concentration solutions (≈30 mL) of certain electrolytes. The produced current and voltage were recorded with a Keithley 6517B Electrometer.

The transference number of cations is calculated with Equation (3)^[^ ^68^ ^]^:

(3) $$t_{+}=\frac{1}{2}\;\left[\frac{E_{\mathrm{diff}}}{\frac{RT}{zF}ln\frac{\gamma_{H}C_{H}}{\gamma_{L}C_{L}}}+1\right]$$

where *E*_diff_ is the diffusion potential, *R*, *T*, z, *F*, and γ, gas constant, temperature, charge number, Faraday constant, and mean activity coefficient, respectively. *C*_H_ and *C*_L_ are the high and low concentrations of the electrolytes.

The efficiency is calculated with Equation (4) as:

(4) $$\eta_{\max}=\frac{{\left(2t_{+}-1\right)}^{2}}{2}$$

The maximum power density in the membranes is calculated with Equation (5) as^[^ ^84^ ^]^:

(5) $$P_{\max}=\frac{V_{\mathrm{OCP}}\times I_{\mathrm{SC}}}{4A}$$

where *V*_OCP_ is the Open circuit potential, *I*_SC_ the short circuit current, and *A* the effective working area, in this case 0.03 mm^2^.

For the resistance calculations, the output power density (*P*_out_) can be estimated with Equation (6)^[^ ^20^ ^]^:

(6) $$P_{\mathrm{out}}=I_{\mathrm{load}}^{2}\times R_{\mathrm{load}}$$

where *I*_load_ is the current corresponding to the connected external resistor *R*_load_.

### Statistical Analysis

Each experiment was repeated at least three times for the energy harvesting measurements. Pre‐processing of data included evaluation of outliers. The magnitude of the corresponding variable is expressed as the mean and the standard deviation (SD), for at least two measurements (*n* ≥ 2). Statistical analysis was executed using Origin software.

## Conflict of Interest

The authors declare no conflict of interest.

## Supporting information

Supporting Information

## Data Availability

The data that support the findings of this study are available from the corresponding author upon reasonable request.
