# Supplementary material for: Optimizing Nanofluidic Energy Harvesting in Synthetic Clay‐based Membranes by Annealing Treatment
Source: Adv Sci (Weinh). 2024 Jun 17;11(31):2400233. doi: 10.1002/advs.202400233 (PMC11336939; doi:10.1002/advs.202400233)
Supplement: Supplementary file 1 — Supporting Information [file ADVS-11-2400233-s001.pdf]

ADVANCED SCIENCE
Open Access

Supporting Information

for *Adv. Sci.*, DOI 10.1002/advs.202400233

Optimizing Nanofluidic Energy Harvesting in Synthetic Clay-based Membranes by Annealing Treatment

Yozelin Zavala-Galindo, Guoliang Yang, Hanwen Zang, Weiwei Lei and Dan Liu**

Supplementary Information

Optimizing Nanofluidic Energy Harvesting in Synthetic Clay-based Membranes by Annealing Treatment

Yozelin Zavala-Galindo, Guoliang Yang, Hanwen Zang, Weiwei Lei, Dan Liu*.*

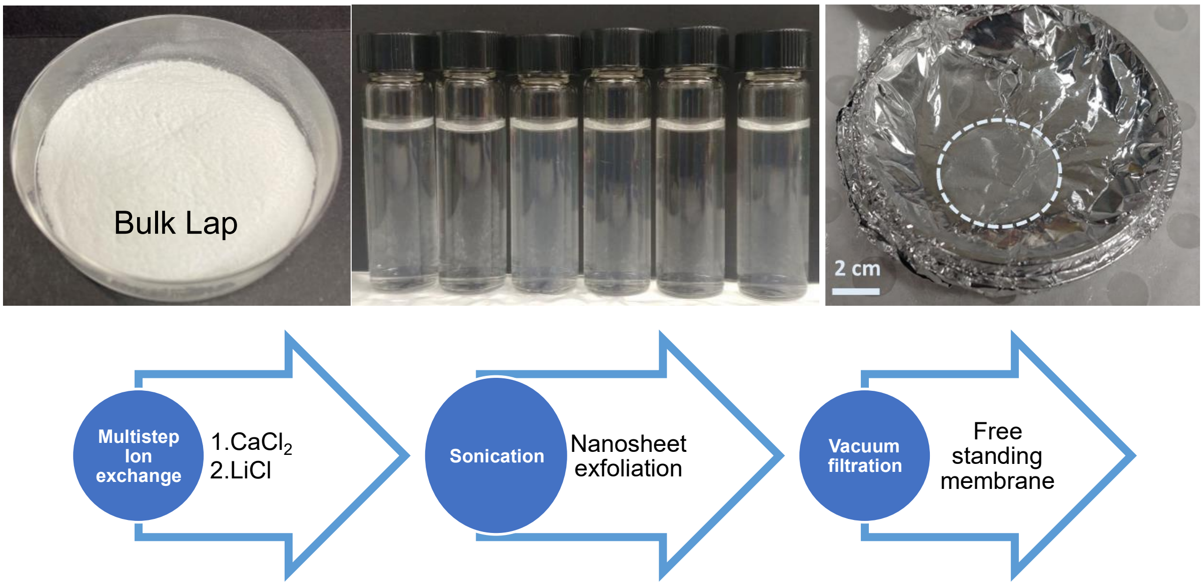

Figure S1. Schematic representation of the Laponite® exfoliation process.

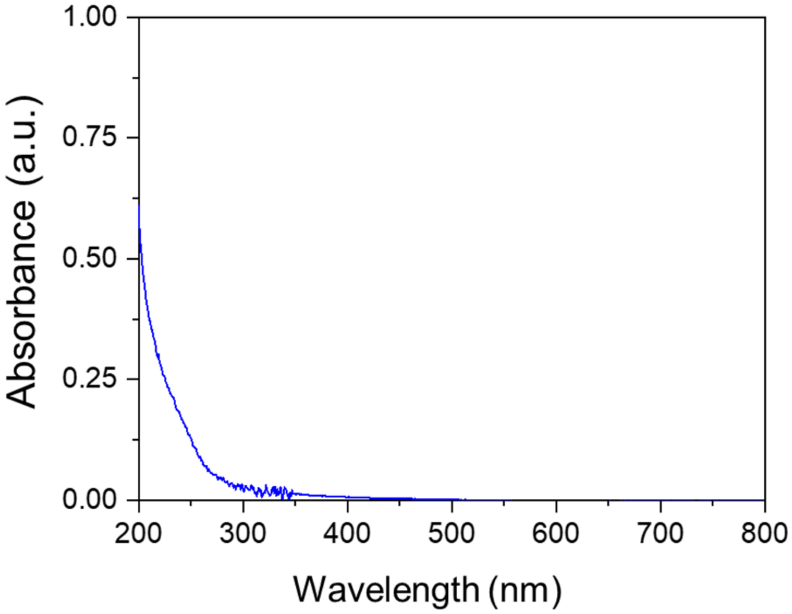

Figure S2. Representative UV spectrum of exfoliated Lap-nanosheets dispersion.

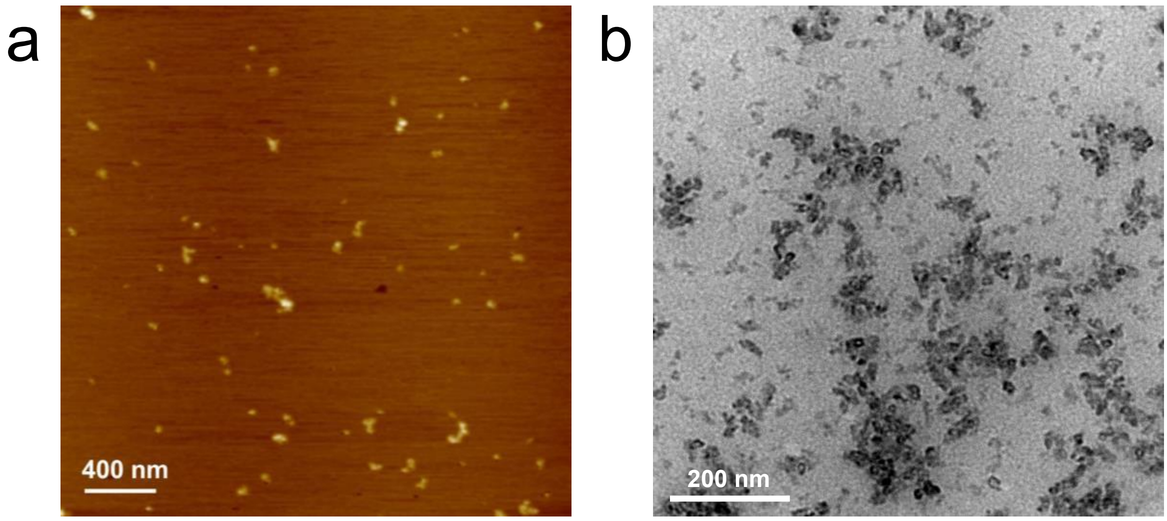

Figure S3. Morphology of nanosheets **(a)** AFM of Lap-nanosheets **(b)** TEM of Lap-nanosheets.

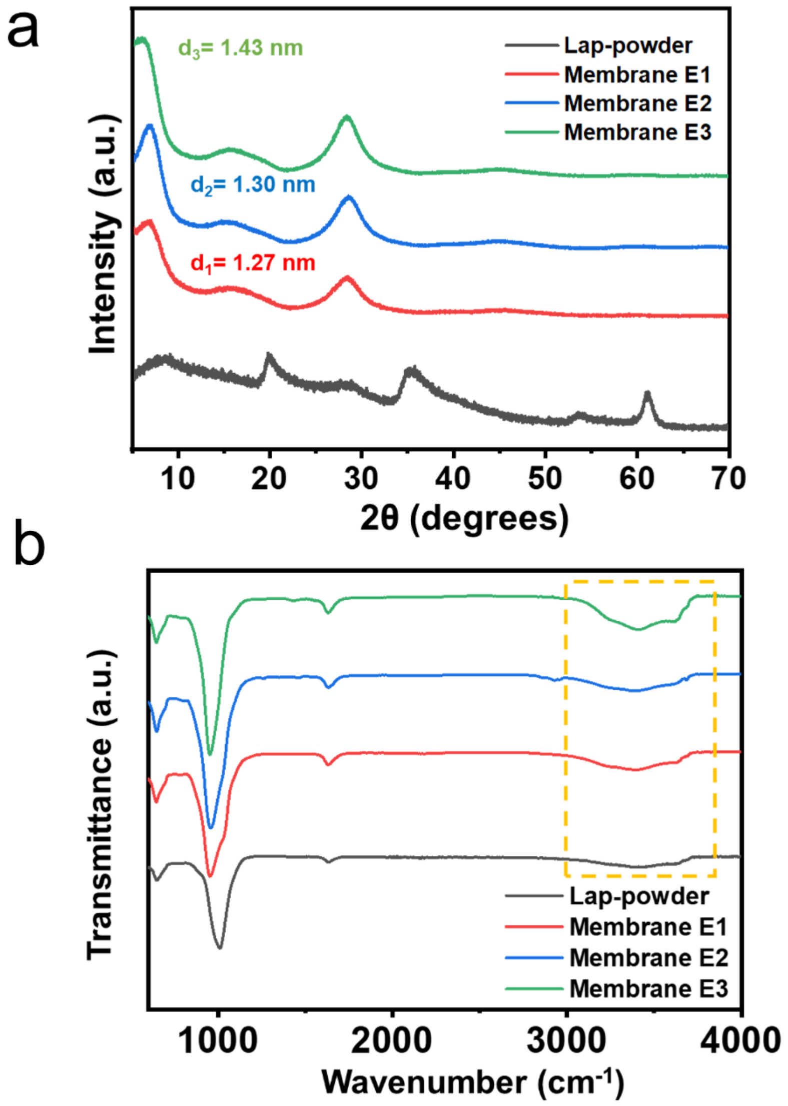

Figure S4. (a) XRD and **(b)** FTIR of bulk powder and reassembled Lap-membranes with different content of absorbed water after vacuum filtration. (**E$_x$** x-represents the filtration experiment).

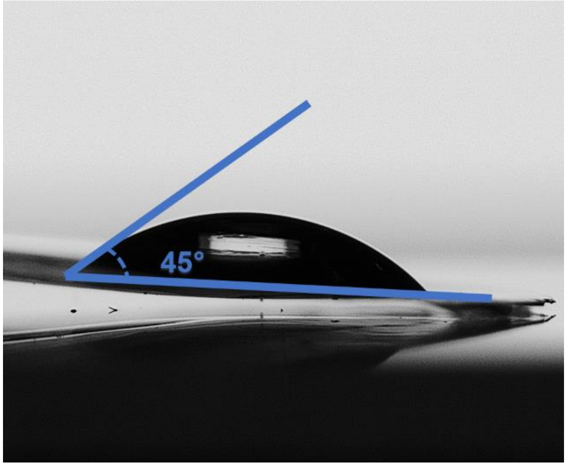

Figure S5. Water contact angle for a reassembled Lap-membrane displaying the high hydrophilic property of the membrane.

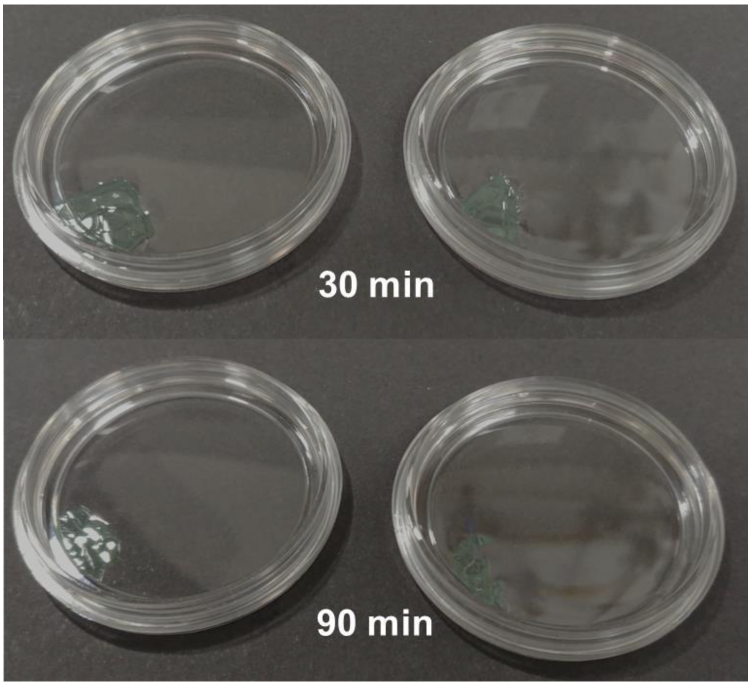

Figure S6. Water stability test for pristine Lap-membrane in DI water. At 30 minutes the membrane is fully hydrated, and it starts delaminating in less than 2 hours. **Note: membranes have been digitally coloured for clarity.*

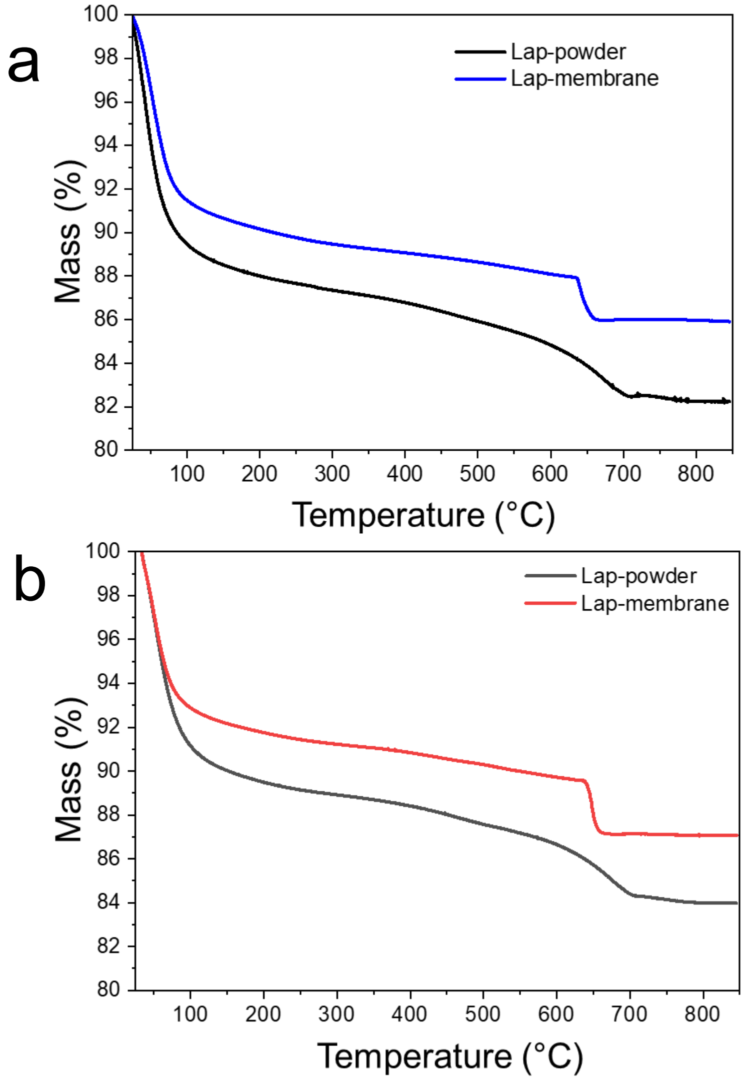

Figure S7. Representative thermogravimetric analysis of Lap-powder and Lap-membranes under **(a)** oxygen atmosphere **(b)** nitrogen atmosphere.

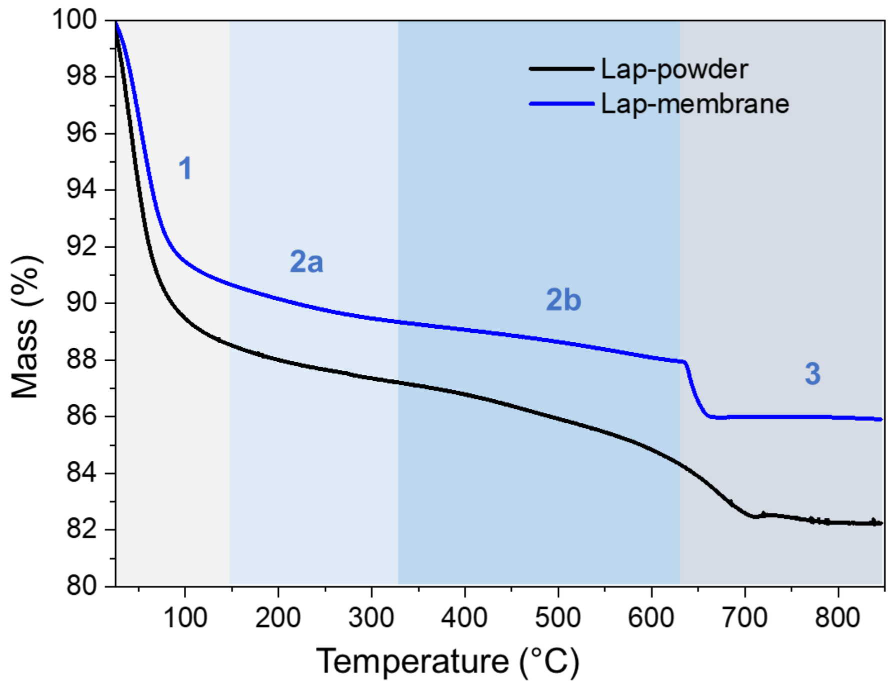

Figure S8. Thermogravimetric analysis of a Lap-membrane and Lap-powder under oxygen atmosphere.

Three zones can be identified associated with the causes of mass loss: **1.** Evaporation of physically absorbed water (superficial water) **2.** Water removal from interlayer **a)** bound water **b)** structural water **3.** Exothermic phase transition and beginning of –OH decomposition.[1]

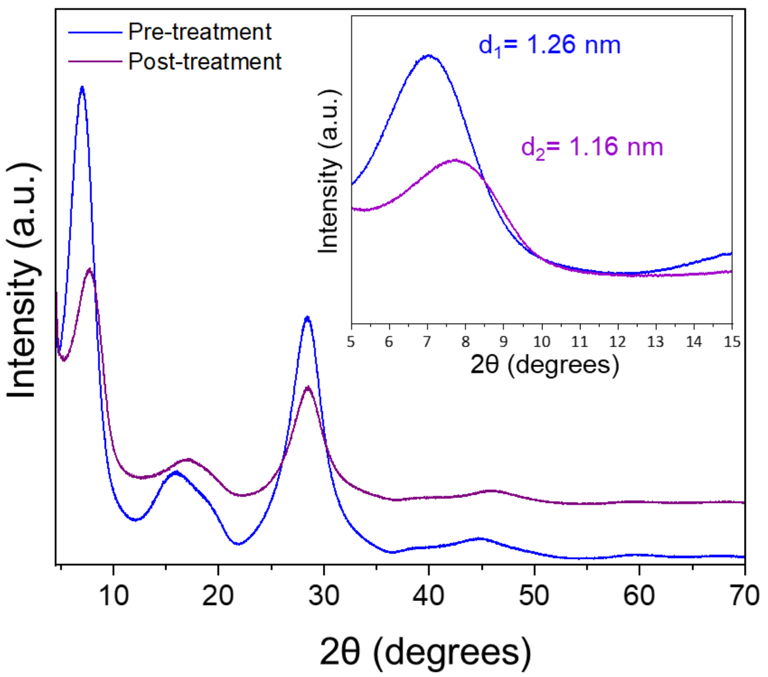

Figure S9. XRD of Lap-membrane before and after annealing in a conventional oven at 180°C for 3 days.

a

b

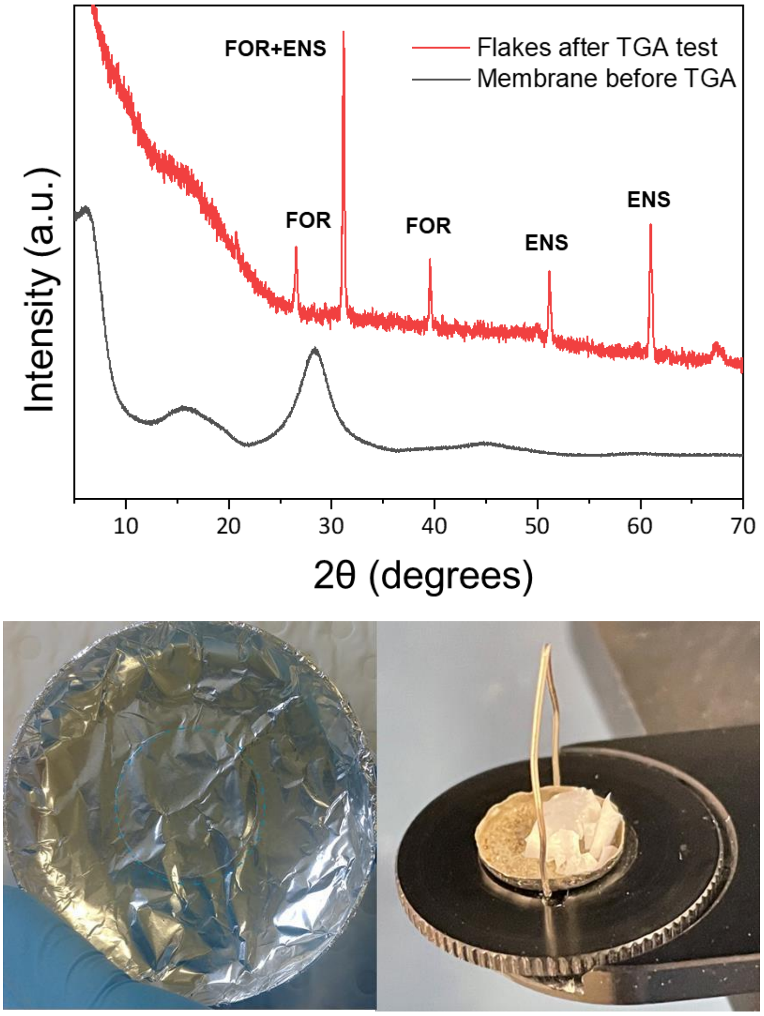

Figure S10. (a) XRD of a Lap-membrane before and after TGA. Amorphous structure changes to Enstatite $MgSiO_3$ (ENS) and Forsterite Mg_2SiO_4 (FOR) phases after 650°C.[2] **(b)** the phase change was accompanied by a change in colour and texture in the membranes.

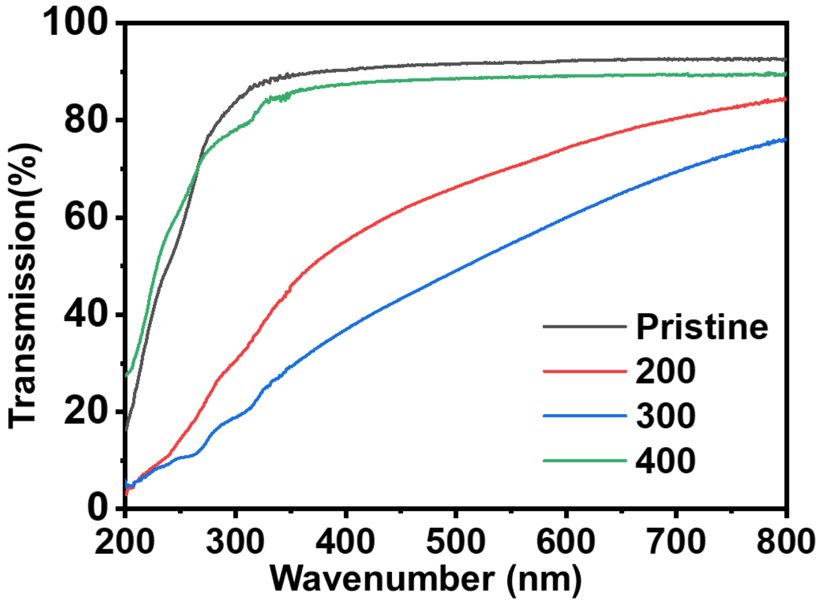

Figure S11. UV-Vis spectra of Lap-membranes. An intermediate structural is suggested with the variation on the optical properties of the membranes. While the pristine membrane and the 400-membranes have a prominent shift near the ultraviolet range, the step is smoother for the 200-membrane and the transmission has almost a linear pattern for the 300-membrane.

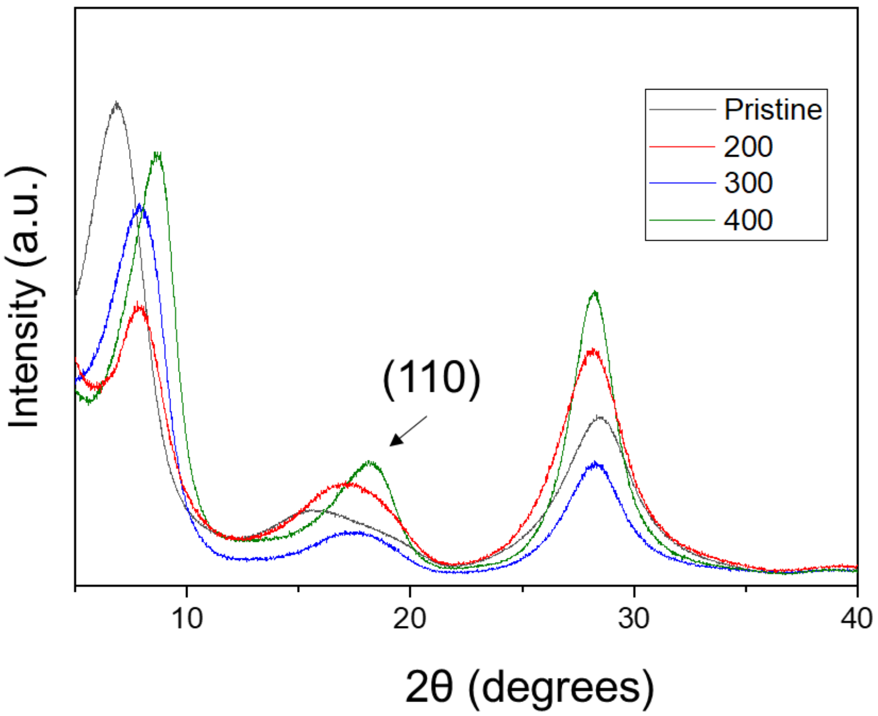

Figure S12. XRD of Lap-membranes. Number indicates the temperature of annealing treatment in °C.

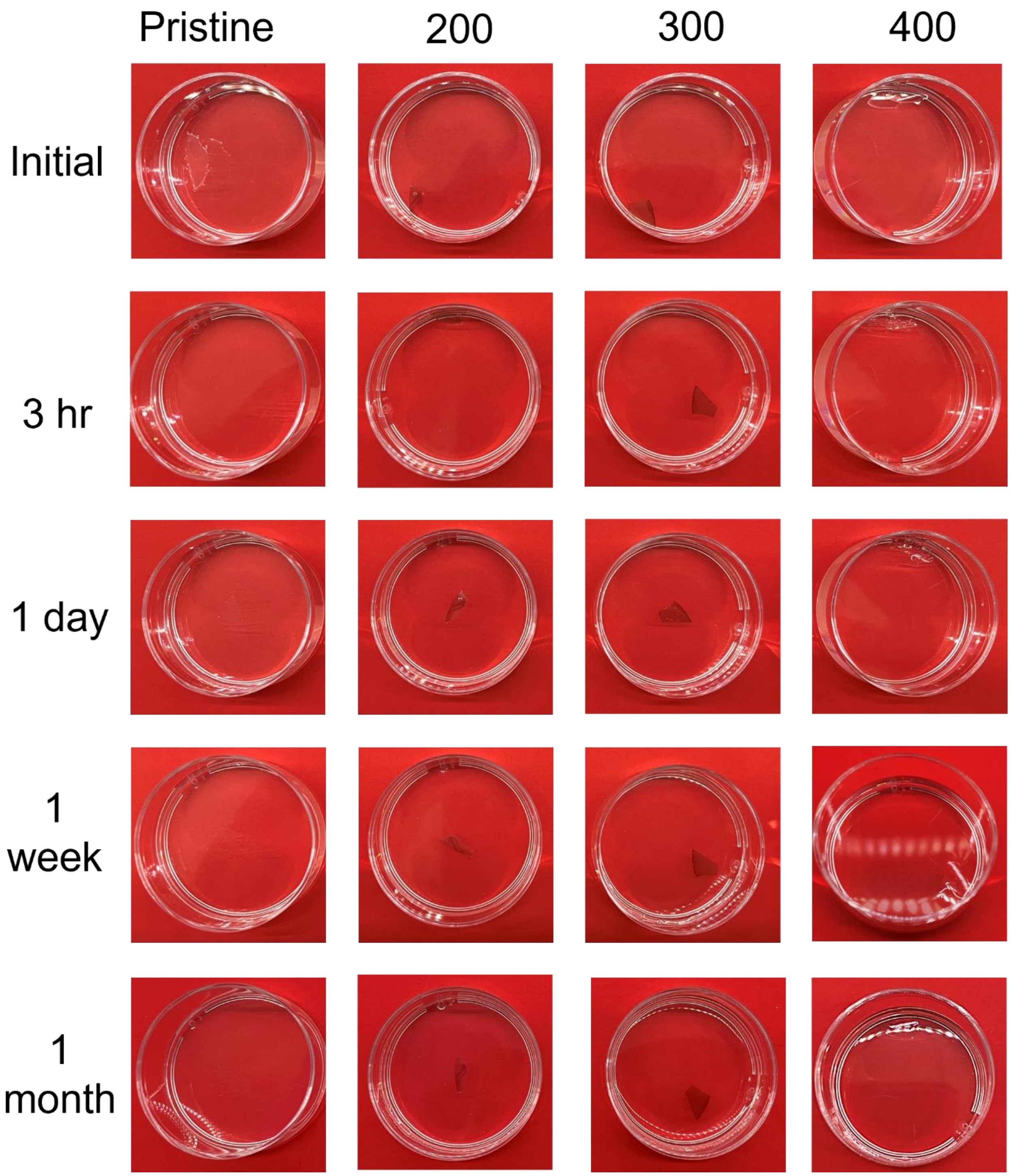

Figure S13. Water stability in DI water for the different Lap-membranes.

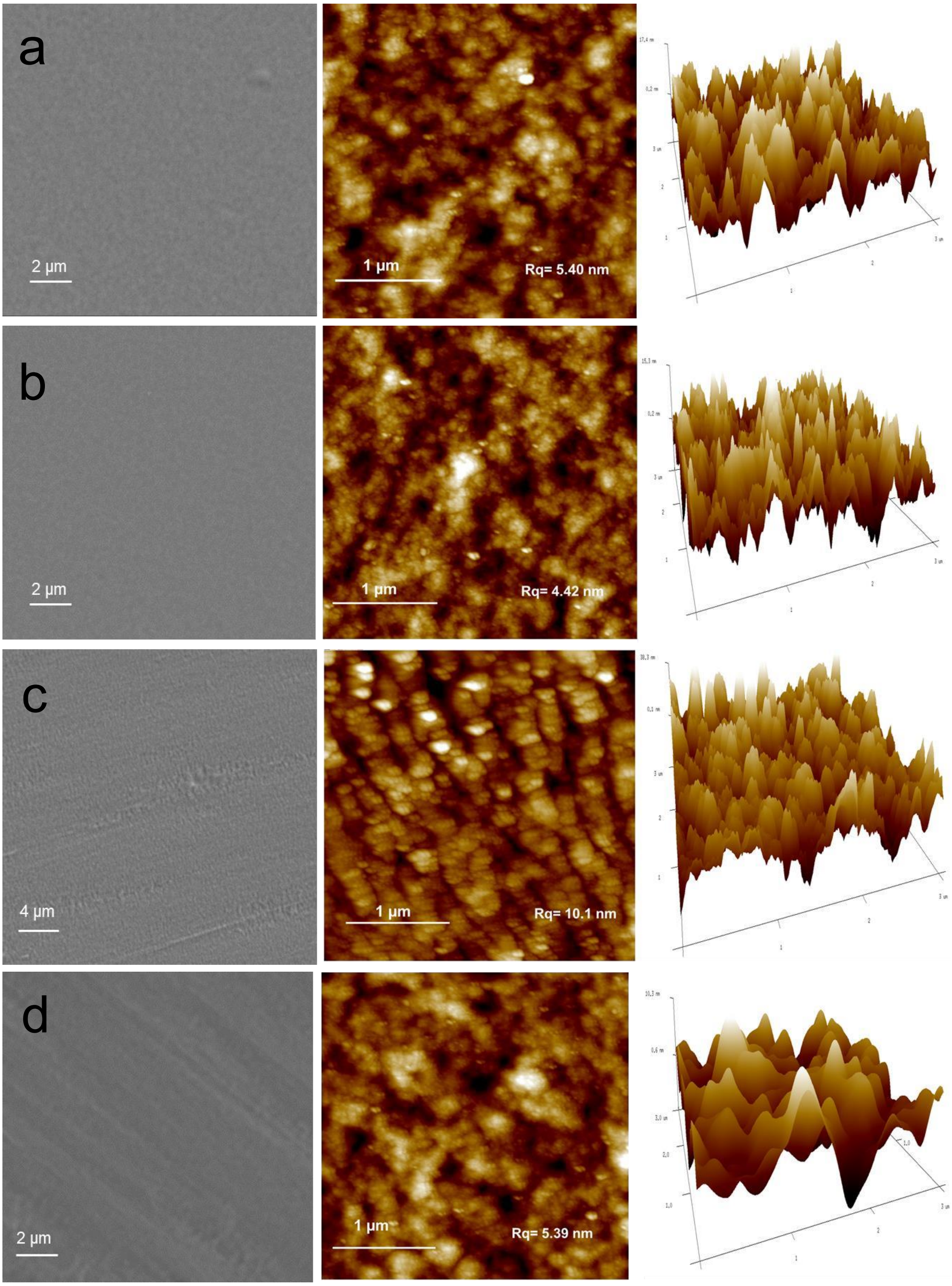

Figure S14. Top-view SEM images, AFM images and their corresponding 3D AFM topographies of **(a)** Pristine **(b)** 200 **(c)** 300 **(d)** 400 membranes, respectively.

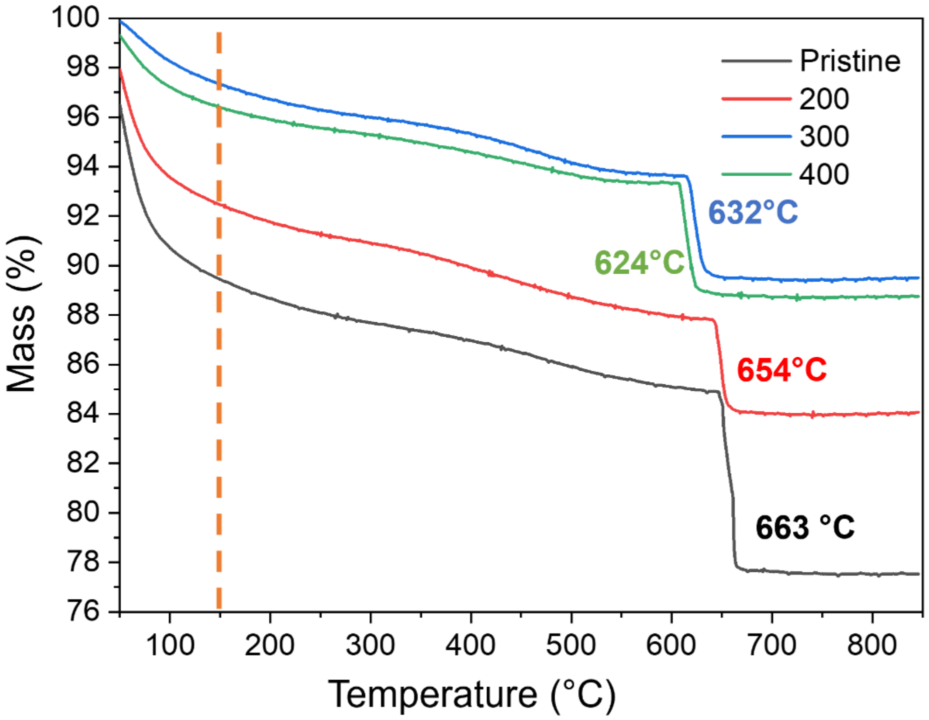

Figure S15. Thermogravimetric analysis of treated Lap-membranes under oxygen atmosphere. “Pristine” denominates the membrane without thermal treatment before TGA.

Table S1. Mass loss analysis per zone.

Sample	Mass change (t<150°C)	Change in Zone 2 (water removal from interlayer)	Total Change before phase transition	Change at transition	Phase transition (°C)	Total mass change
**Pristine**	7.3%	**4.7%**	12.0%	7.7%	663	19.7%
**200**	5.6%	**4.9%**	10.5%	3.7%	654	14.2%
**300**	2.5%	**3.8%**	6.3%	4.1%	632	10.4%
**400**	3.0%	**3.0%**	6.0%	4.6%	624	10.6%

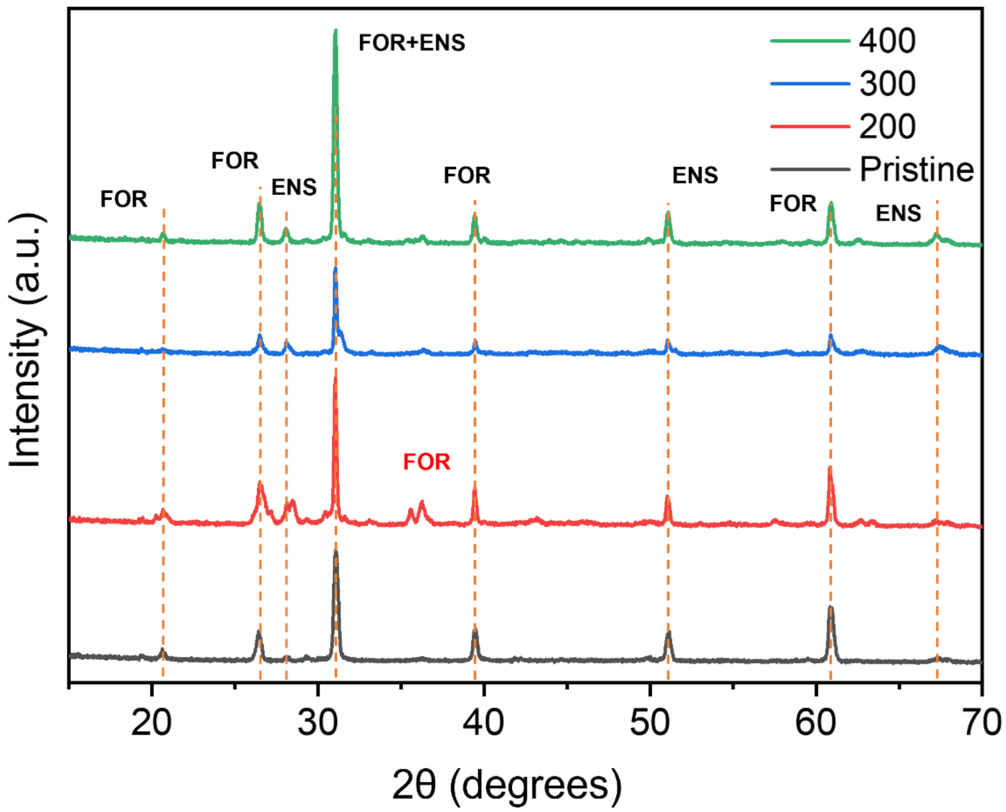

Figure S16. XRD of treated Lap-membranes after TGA. Amorphous structure changes to Enstatite $MgSiO_3$ (ENS) and Forsterite Mg_2SiO_4 (FOR) phases in distinct distributions.

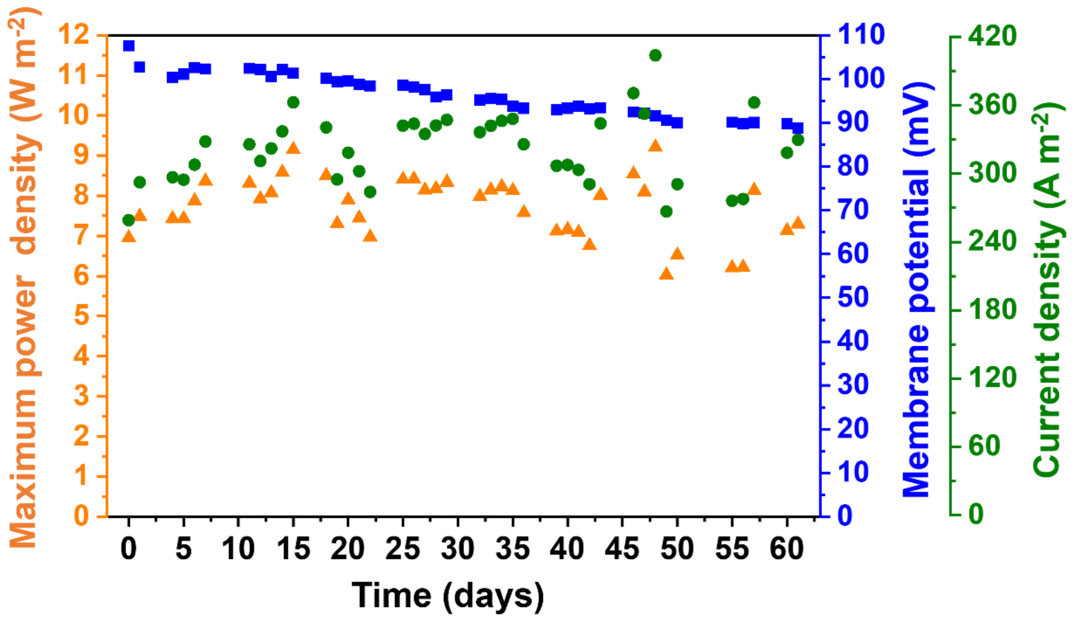

Figure S17. Long term maximum power density, current density and membrane potential for the 300-membrane at 30-fold (C_H/C_L=3 м/0.1 м) NaCl.

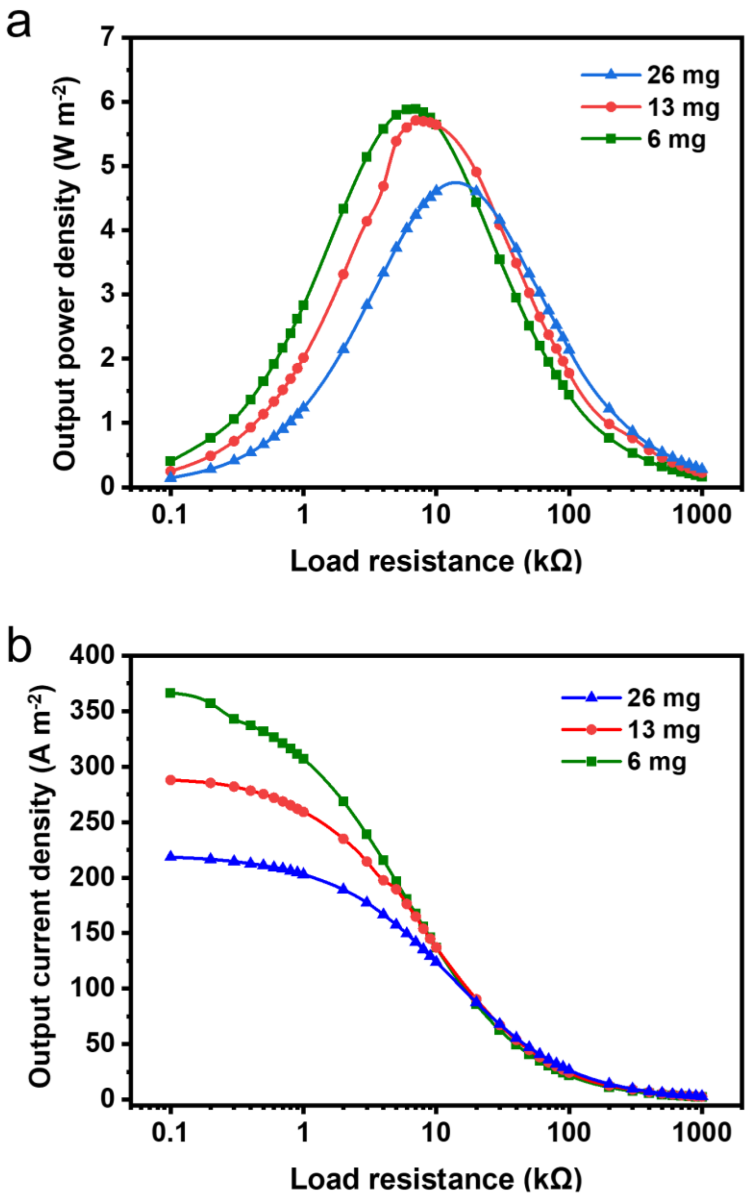

Figure S18. (a) Output power density and **(b)** output current density of the Lap-membranes with different nanosheet contents as functions of load resistance.

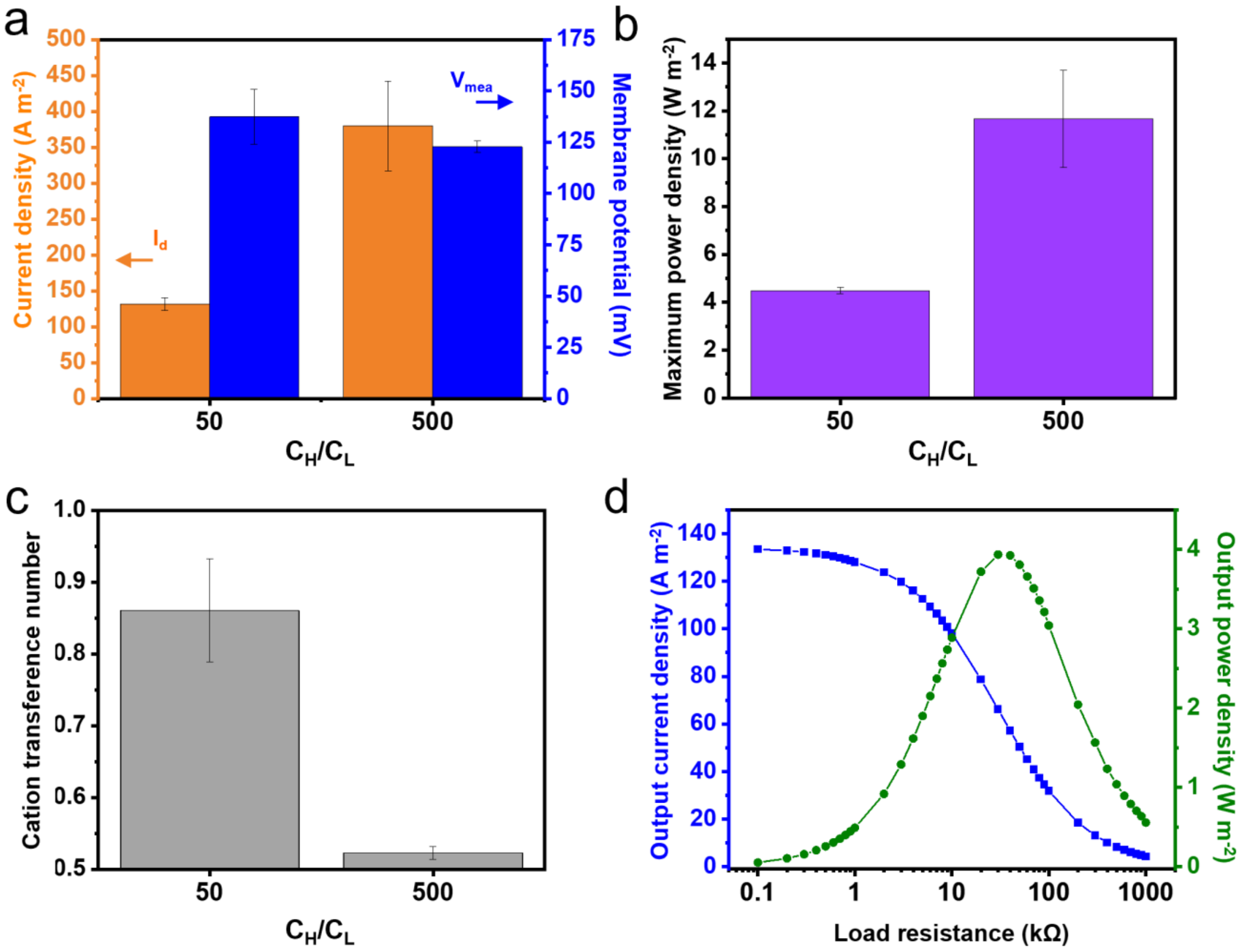

Figure S19. (a)-(c) Current density, membrane potential, maximum power density and cation transfer number under hyper salinity and artificial sea water concentration gradients 500-fold and 50-fold (C_H/C_L, C_L fixed to 10mM) for the thermally treated membranes at 300°C (mean ± SD, n = 3). **(d)** Output current density and output power density as function of load resistance at the 50-fold.

Table S2. Output power density and efficiency of representative membranes in the literature.

Membrane types	Maximum power density ($W\ m^{-2}$)	Energy conversion efficiency (%)	Concentration gradient	Electrolyte
**Graphene oxide pairs**[3]	0.77	36.6	0.5 M/10 mM	NaCl
**MXene/Kevlar**[4]	3.7	35	0.5 M/10 mM	NaCl
**Graphene oxide**[5]	4.1	35	0.5 M/10 mM	NaCl
**Graphene oxide/Silk**[6]	5.07	27.2	0.5 M/10 mM	NaCl
**Silk-based**[7]	2.43	17.2	0.5 M/10 mM	NaCl
**Kaolinite based**[8]	0.18	44.2	1 M/10 mM	KCl
**Molybdenum disulfide**[9]	5.2	32	0.5 M/10 mM	NaCl
**Montmorillonite based**[10]	4.1	18	0.5 M/10 mM	NaCl
**Bacterial Cellulose/ Graphene oxide**[11]	4.86	32	0.5 M/10 mM	NaCl
**Montmorillonite based**[12]	0.02	35.3	10 mM/1 mM	KCl
**MXene**[13]	4.6	44.2	0.5 M/10 mM	NaCl
**LDH/AAO**[14]	2.85	17.6	0.5 M/10 mM	NaCl
**Graphene oxide/Cellulose**[15]	4.19	30	0.5 M/10 mM	KCl
**Montmorillonite/ Aramid Nano Fiber** [16]	3.6	16	100 mM/0.01 mM	KCl
**Vermiculite**[17]	10.9	20.7	1 M/1 mM	KCl
**Laponite® (this work)**	9.89	23.1	3 M /100 mM	NaCl
**Laponite® (this work)**	4.5	27	0.5 M/10 mM	NaCl

References

[1] Y. Liu, X. Ding, L. Chen, W. Tian, X. Xu, K. Zhang, *Inorg Chem* **2023**, *62*, 5400.

[2] L. M. Anovitz, A. J. Rondinone, L. Sochalski-Kolbus, J. Rosenqvist, M. C. Cheshire, *J Colloid Interface Sci* **2017**, *495*, 94.

[3] J. Ji, Q. Kang, Y. Zhou, Y. Feng, X. Chen, J. Yuan, W. Guo, Y. Wei, L. Jiang, *Adv Funct Mater* **2017**, *27*, DOI 10.1002/adfm.201603623.

[4] Z. Zhang, S. Yang, P. Zhang, J. Zhang, G. Chen, X. Feng, *Nat Commun* **2019**, *10*, DOI 10.1038/S41467-019-10885-8.

[5] Y. Qian, D. Liu, G. Yang, L. Wang, Y. Liu, C. Chen, X. Wang, W. Lei, *J Am Chem Soc* **2022**, *144*, 13764.

[6] W. Xin, H. Xiao, X.-Y. Kong, J. Chen, L. Yang, B. Niu, Y. Qian, Y. Teng, L. Jiang, L. Wen, **2020**, *27*, 53.

[7] W. Xin, Z. Zhang, X. Huang, Y. Hu, T. Zhou, C. Zhu, X.-Y. Kong, L. Jiang, L. Wen, *Nat Commun* **2019**, *10*, DOI 10.1038/s41467-019-11792-8.

[8] H. Cheng, Y. Zhou, Y. Feng, W. Geng, Q. Liu, W. Guo, L. Jiang, *Advanced Materials* **2017**, *29*, 1.

[9] C. Zhu, P. Liu, B. Niu, Y. Liu, W. Xin, W. Chen, X. Y. Kong, Z. Zhang, L. Jiang, L. Wen, *J Am Chem Soc* **2021**, *143*, 1932.

[10] Z. Ding, T. Gu, S. Sun, G. Tang, H. Zhang, T. Wang, Y. Luo, J. Li, **2023**, DOI 10.1039/d2ta08991b.

[11] N. Sheng, M. Zhang, Q. Song, H. Zhang, S. Chen, H. Wang, K. Zhang, *Nano Energy* **2022**, *101*, 107548.

[12] Y. Zhou, H. Ding, A. T. Smith, X. Jia, S. Chen, L. Liu, S. E. Chavez, Z. Hou, J. Liu, H. Cheng, Q. Liu, L. Sun, *J Mater Chem A Mater* **2019**, *7*, 14089.

[13] L. Ding, D. Xiao, Z. Lu, J. Deng, Y. Wei, J. Caro, H. Wang, *Angewandte Chemie - International Edition* **2020**, *59*, 8720.

[14] Y. Liu, J. Ping, Y. Ying, **2022**, DOI 10.1002/advs.202103696.

[15] Y. Wu, W. Xin, X.-Y. Kong, J. Chen, Y. Qian, Y. Sun, X. Zhao, W. Chen, L. Jiang, L. Wen, *2702 | Mater. Horiz* **2020**, *7*, 2702.

[16] R. Qin, J. Tang, C. Wu, Q. Zhang, T. Xiao, Z. Liu, Y. Jin, J. Liu, H. Wang, *Nano Energy* **2022**, *100*, 107526.

[17] L. Cao, H. Wu, C. Fan, Z. Zhang, B. Shi, P. Yang, M. Qiu, A. Niaz, A. Khan, Z. Jiang, *J Mater Chem A Mater* **2021**, *9*, 14576.
